# Intraoperative allogeneic blood transfusion is not associated with postoperative acute kidney injury and in-hospital mortality in liver transplantation patients: a propensity score matching analysis

**DOI:** 10.3389/fmed.2026.1748464

**Published:** 2026-06-23

**Authors:** Yi Wang, Zhipeng Wu, Man Lai, Huihui Zhang, Ying Xu, Yanmei Gu, Qinwei Yao, Guangming Li, Yingmin Ma, Xin Wang

**Affiliations:** 1 Beijing Institute of Hepatology, Beijing Youan Hospital, Capital Medical University, Beijing, China; 2 Department of Intensive Medicine, Beijing Youan Hospital, Capital Medical University, Beijing, China; 3 Department of Respiratory and Critical Care Medicine, Beijing Youan Hospital, Capital Medical University, Beijing, China; 4 Department of General Surgery, Beijing Youan Hospital, Capital Medical University, Beijing, China

**Keywords:** acute kidney injury, allogeneic blood transfusion, in-hospital mortality, intraoperative, liver transplantation, postoperative, propensity score matching

## Abstract

**Background:**

Many studies have found a correlation between perioperative blood transfusion and postoperative complications and mortality in liver transplant (LT) patients. The purpose of our study is to identify independent risk factors for intraoperative blood transfusion in liver transplant patients and to explore the effect of intraoperative blood transfusion on postoperative acute kidney injury (AKI) and in-hospital mortality in LT patients.

**Methods:**

A total of 686 liver transplant patients were included in the study, of which 517 (75.4%) patients received an intraoperative blood transfusion (IBT group). Univariable and multivariable regression were used to identify independent risk factors for IBT, and propensity score matching (PSM) and multivariable regression analysis were used to analyze the effect of IBT on postoperative AKI and in-hospital mortality.

**Results:**

The multivariable regression analysis revealed that sex (*p* = 0.044), weight (*p* = 0.005), liver cancer (*p* = 0.003), alcoholic hepatitis (*p* = 0.048), Child-Pugh classification (*p* = 0.006), anhepatic phase (*p* = 0.044), and intraoperative bleeding (*p* < 0.0001) were independent risk factors for IBT. In the unmatched cohort, IBT was significantly associated with an increased risk of post-LT AKI (OR 2.993, 95% CI 2.061–4.347, *p* < 0.0001) and in-hospital mortality (OR 2.692, 95% CI 1.313–5.522, *p* = 0.007). After multivariable logistic regression, IBT remained an independent risk factor for both post-LT AKI and in-hospital mortality in the unmatched cohort. After propensity score matching, 105 pairs of patients were obtained. Among the 105 patients who received IBT, the vast majority, 97 patients (92.4%), received no more than 8 units of blood. In the matched cohort, IBT was significantly associated with an increased risk of post-LT AKI (OR: 1.861, 95% CI: 1.053–3.290, *p* = 0.033), but not associated with in-hospital mortality (OR: 1.000, 95% CI: 0.281–3.562, *p* = 1.000). After multivariable regression analysis, IBT was no longer an independent risk factor for post-LT AKI and hospital mortality in the matched cohort.

**Conclusion:**

In our matched cohort, the vast majority of patients, received no more than 8 units of blood. Intraoperative allogeneic blood transfusion does not influence postoperative acute kidney injury and in-hospital mortality in liver transplantation patients. Multicenter, prospective studies are needed in the future to validate our findings.

**Clinical trial registration:**

CHiCTR1900024561; https://www.chictr.org.cn/showproj.html?proj=41126.

## Introduction

Liver transplantation is the last resort for many patients with irreversible liver disease (1, 2). This is a complex surgery that requires the efforts and coordination of multiple disciplines. The surgery involves major vascular procedures, and patients may experience coagulation dysfunction, which can lead to significant bleeding and unstable blood pressure during the surgery (3, 4). Therefore, blood transfusion is a common practice in liver transplant patients as an important means of maintaining oxygen delivery and improving coagulation function (5, 6).

However, transfusion may also bring a range of adverse prognostic factors, such as transfusion reactions, immunosuppression and increased circulatory load from over-transfusion (7–12). Many studies have indicated that perioperative transfusion is associated with postoperative complications and mortality (13–24), and is an independent predictor of postoperative death in liver transplant surgery (19, 20, 23). Early allograft dysfunction (EAD) and postoperative acute kidney injury (AKI) are common postoperative complications in liver transplant patients and are associated with patient mortality (25–28). Some studies have suggested a correlation between blood transfusion and the incidence of EAD and postoperative AKI (13, 16, 29).

Certainly, as a liver transplant physician, reducing bleeding during surgery is certainly something we strive for. However, intraoperative blood loss can be influenced by many factors, such as patient age, severity of illness, and technical difficulties in achieving hemostasis due to inexperienced surgical staff. Non-surgical factors contributing to blood loss include coagulation disorders caused by deficiencies in clotting factors, reduced platelet count, and blood transfusion may become necessary (21, 24). Therefore, we hypothesize that the effect of intraoperative transfusion on prognosis may be due to these confounding variables rather than the transfusion itself. However, due to the potential violation of medical ethics principles, it is not possible to conduct randomized controlled trials (RCTs) to address this issue. Previous studies have relatively small sample sizes (13–15, 19), and the transfusion during the entire perioperative period was taken into consideration (15, 16, 22). We want to evaluate only the impact of allogeneic red blood cell transfusion during the intraoperative period on postoperative AKI and in-hospital mortality rate. To elucidate the relationship between intraoperative transfusion and postoperative AKI and in-hospital mortality rate in liver transplant patients, we conducted a retrospective study using propensity score matching and multivariable logistic regression analysis.

## Patients and methods

**Figure 1:**
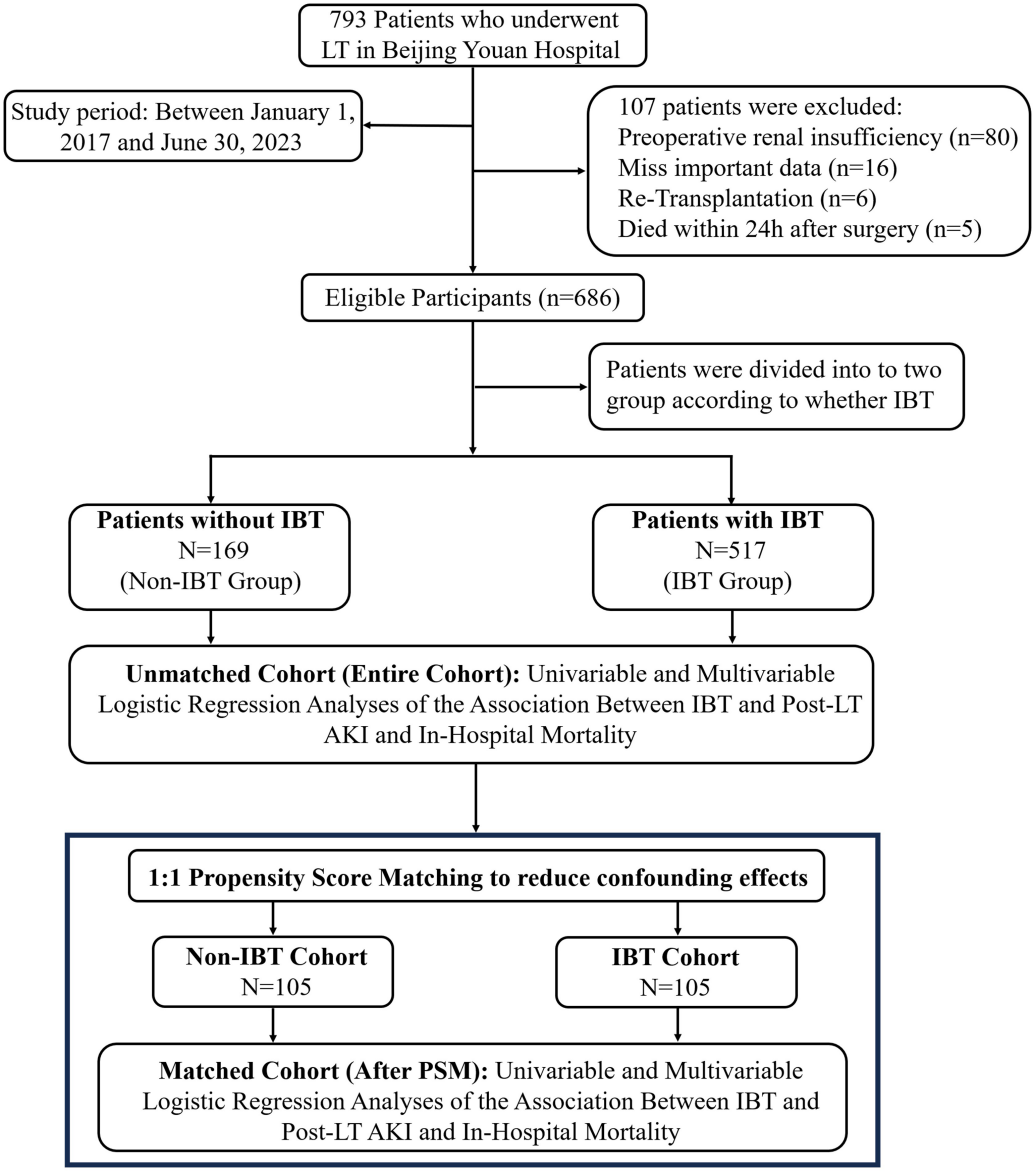
Flow diagram of patient enrollment and data analysis. LT, liver transplantation; IBT, intraoperative blood transfusion; PSM, propensity score matching.

793 patients who underwent LT at the Department of General Surgery in Beijing Youan Hospital between January 1, 2017 and June 30, 2023 were all screened. Clinical information was extracted from the electronic medical record system. We excluded patients with preoperative renal insufficiency, re-transplantation, death within 24 h after surgery, missing important data, or age <18 years. And finally, in total 686 patients were included (Figure 1). All LT recipients were registered with the China Organ Transplant Response System. This study involving human participants was reviewed and approved by the Ethics Committee of Beijing Youan Hospital, Capital Medical University (approval number: [2019] 016). The study was conducted in accordance with the Declaration of Helsinki and relevant national and institutional guidelines. Given the retrospective design and use of anonymized data, the requirement for written informed consent was waived by the Ethics Committee. All patient data were anonymized and handled with strict confidentiality. This clinical study was registered in the Chinese Clinical Trial Register (CHiCTR1900024561; https://www.chictr.org.cn/showproj.html?proj=41126).

### Preoperative evaluation

Preoperative preparations for liver transplantation typically include the following: Physical examination, blood tests, including liver function, electrolytes, complete blood cell count, and coagulation function, urine test, Electrocardiogram and chest Computed Tomography (CT) scan, abdominal ultrasound or CT scan, electroencephalogram and neuropsychological evaluation (used to assess hepatic encephalopathy), hepatic virology and immunology tests, cardiac evaluation and pulmonary function tests, in addition, specific tests and evaluations may be performed based on the patient’s individual circumstances. Anesthesia evaluation and blood product preparation.

### Surgical procedures

Orthotopic liver transplantation was performed by experienced senior transplant surgeons according to our institutional protocol. Most procedures used the piggyback technique with preservation of the recipient’s retrohepatic inferior vena cava, followed by end-to-end reconstruction of the portal vein, hepatic artery, and bile duct. In a minority of patients with tumor invasion or unfavorable caval anatomy, a conventional caval replacement technique was applied, with resection of the retrohepatic inferior vena cava together with the native liver and replacement by the donor inferior vena cava. All patients underwent standard intraoperative monitoring, including continuous arterial blood pressure and central venous pressure measurements. Intraoperative transfusion decisions were made by the attending anesthesiologist in consultation with the surgeon, based on hemoglobin levels, hemodynamic status, and estimated blood loss. Patients with hepatocellular carcinoma did not receive autologous blood reinfusion, and only the volume of allogeneic red blood cell transfusion was recorded for this study.

### Postoperative management

After the surgery, patients were immediately transferred to the intensive care unit for observation, and then transferred to the general ward according to their condition. Routine administration of broad-spectrum antibiotics for postoperative infection treatment. Monitor the patient’s vital signs, observe the appearance and drainage of the incision, observe the patient’s urine output, perform blood tests, and conduct liver and kidney function tests. Observe whether the patient develops complications, such as infection, bleeding, blood clots, and other conditions. Monitor the patient’s nutritional status, including weight, blood protein, serum albumin, and other indicators.

### Variables and outcomes

Clinical information, including demographic data, disease course, medical history, and laboratory test results, was extracted from the institutional electronic medical records system, All the data were entered using “Excel.” Recorded variables were divided into preoperative, intraoperative, and postoperative parameters. The preoperative participant factors included but were not limited to sex, age, body mass index, and primary liver diseases. Intraoperative factors included operative time, and intraoperative hypotension. Postoperative factors included laboratory parameters (e.g., postoperative lactic acid, postoperative alanine aminotransferase [ALT]) and postoperative clinical outcomes (e.g., early allograft dysfunction [EAD] and post - LT AKI). Detailed information can be found in Table 1. The main outcomes of this study were in-hospital mortality and post - LT AKI. We also investigated the association between IBT and postoperative EAD, and we compared postoperative outcomes between the IBT and non-IBT groups, including the need for renal replacement therapy (RRT), duration of mechanical ventilation, re-intubation, and length of ICU stay. Postoperative AKI was diagnosed according to the 2012 Kidney Disease: Improving Global Outcomes (KDIGO) clinical practice guidelines (30, 31): (1) an increase in serum creatinine (sCr) level ≥26.5 μmol/L (0.3 mg/dL) within 48 h; or (2) increase in sCr level to ≥1.5 times the baseline level within 7 days after LT; or (3) urine volume (UV) <0.5 mL/(kg × h) for >6 h post-LT. Intraoperative hypotension was defined as a decrease in systolic blood pressure (SBP) of more than 20% from baseline or an absolute SBP of less than 90 mmHg (32, 33). EAD was defined as the presence of one or more previously established postoperative laboratory criteria reflecting hepatic injury and function (34).”

**Table 1:** Comparisons of patients’ characteristics and operative variables between the non-intraoperative blood transfusion (non-IBT) and IBT groups in the unmatched cohort.

Characteristics	Total (*n* = 686)	Non-IBT (*n* = 169)	IBT (n = 517)	*p*-value
Demographic data				
Age (years)	53 (45,59)	54 (45,59)	53 (45,60)	0.663
Sex, male (*N*, %)	551 (80.3%)	148 (87.6%)	403 (77.9%)	0.011*
Height (cm)	170 (166, 175)	172 (169, 175)	170 (165, 175)	0.045*
Weight (kg)	70 (63, 78)	71 (65, 80)	70 (62,78)	0.023*
BMI (Kg/m^2^)	24.2 (22.0, 26.3)	24.3 (22.1, 27.1)	24.2 (21.9, 26.2)	0.178
Primary liver disease				
Liver cancer (*N*, %)	340 (49.6%)	126 (74.6%)	214 (41.4%)	<0.0001*
Viral B hepatitis (*N*, %)	390 (56.9%)	110 (65.1%)	280 (54.2%)	0.009*
Alcoholic hepatitis (*N*, %)	126 (18.4%)	18 (10.7%)	108 (20.9%)	0.002*
Autoimmune hepatitis (*N*, %)	38 (5.5%)	4 (2.4%)	34 (6.6%)	0.065*
Liver cirrhosis (*N*, %)	612 (89.2%)	146 (86.4%)	466 (90.1%)	0.318
Liver failure (*N*, %)	223 (32.5%)	19 (11.2%)	202 (39.1%)	<0.0001*
Liver complications				
Encephalopathy (*N*, %)	141 (20.6%)	18 (10.7%)	123 (23.8%)	<0.0001*
Ascites (*N*, %)	397 (58%)	66 (39.1%)	331 (64.3%)	<0.0001*
Baseline medical status				
Coronary heart diseases (*N*, %)	12 (1.7%)	5 (3.0%)	7 (1.4%)	0.284
Hypertension (*N*, %)	82 (12.0%)	26 (15.4%)	56 (10.8%)	0.129
Diabetes mellitus (*N*, %)	118 (17.2%)	37 (21.9%)	81 (15.7%)	0.110
Child-Pugh classification (*N*, %)				
A	170 (24.7%)	86 (50.9%)	83 (16.1%)	<0.0001*
B	276 (40.3%)	58 (34.3%)	217 (42.2%)	
C	240 (35.0%)	25 (14.8%)	214 (41.6%)	
MELD score	14(9, 21)	10 (7, 15)	15 (10, 23)	<0.0001*
Preoperative laboratory data				
Serum creatinine (μmol/L)	59 (48, 70)	62 (53, 71)	58 (46, 70)	0.007*
AST (U/L)	47 (32, 80)	41 (29, 74)	49 (33, 82)	0.021*
TB (μmol/L)	53 (25, 194)	27 (16, 59)	67 (32, 267)	<0.0001*
Intraoperative details				
Cold ischemia time (h)	5 (5, 6)	5 (4.0, 5.5)	5 (5, 6)	0.845
Anhepatic phase (minute)	52 (46, 65)	49 (44, 56)	55 (47, 66)	<0.0001*
Surgical approach, classic, (*N*, %)	584 (86.9%)	160 (95.8%)	424 (84.0%)	<0.0001*
Operative time (h)	7 (6.09, 7.75)	6.25 (5.45, 7.25)	7.04 (6.19, 8.00)	<0.0001*
Intraoperative bleeding (mL)	1,000 (600, 1,600)	600 (400, 800)	1,200 (700, 1,800)	<0.0001*
Blood product transfusions (mL)	800 (400, 1,600)	0 (0,0)	1,200 (800, 2,000)	<0.0001*
Intraoperative fluid balance (mL)	4,022 (3,100, 5,000)	3,200 (2,540, 3,900)	4,310 (3,500, 5,220)	<0.0001*
Intraoperative hypotension (*N*, %)	158 (23.1%)	19 (11.2%)	139 (26.9%)	<0.0001*
Intraoperative urine output (mL)	1,090 (800, 1,400)	1,100 (840, 1,410)	1,090 (800, 1,400)	0.478

Non-normally distributed continuous variables are displayed as a median with interquartile range (IQR) and were compared using the Mann–Whitney *U* test. Categorical variables are expressed as counts with percentages and were compared using Pearson’s chi–square or Fisher’s exact test. Multiple samples are compared using the non-parametric Kruskal–Wallis test. IBT: intraoperative blood transfusion; BMI: body mass index; MELD: model for end-stage liver disease; AST: aspartate aminotransferase; TB: serum total bilirubin. *p-value < 0.05.

### Propensity score matching

In order to control for biases that may arise from covariables between the two groups of patients, the propensity score matching method (35, 36) was used to match patients in the IBT-group and non-IBT group. This method was implemented using R software (version 2.15; R Foundation for Statistical Computing). Twenty preoperative and intraoperative covariables were used for propensity score matching, including: gender, BMI, etiology (hepatocellular carcinoma, hepatitis B virus, alcoholic liver disease, autoimmune hepatitis, liver cirrhosis, liver failure), hepatic encephalopathy, ascites, coronary heart disease, hypertension, diabetes, Child-Pugh score, MELD score, preoperative creatinine, preoperative total bilirubin, surgical duration, intraoperative bleeding, and intraoperative hypotension. Non-IBT and IBT group patients were matched in a 1:1 ratio, pairing two patients with the closest propensity scores, and without replacement, to minimize conditional bias. We attempted matching with caliper values of 0.2, 0.02, and 0.002, and ultimately found that a caliper value of 0.02 ensured homogeneity while retaining the maximum number of cases possible. We used propensity score matching (PSM) to create comparable IBT and non-IBT groups. Twenty preoperative and intraoperative covariates were included in the propensity score model, and 1:1 nearest neighbor matching without replacement was performed. PSM was chosen over inverse probability of treatment weighting (IPTW) because our primary aim was to construct an easily interpretable matched cohort and, in our data, 1:1 matching already achieved satisfactory covariate balance, with the vast majority of standardized mean differences (SMDs) below 0.1. After propensity score matching, 105 pairs of patients were obtained. Among the 105 patients who received IBT, the vast majority, 97 patients (92.4%), received no more than 8 units of blood.

### Statistical analysis

The normality of continuous variables was assessed using the Shapiro–Wilk test. Normally distributed continuous variables are displayed as mean ± standard deviation (SD) and were compared using the independent-samples Student’s *t* test. Non-normally distributed continuous variables are displayed as median with interquartile range (IQR) and were compared using the Mann–Whitney *U* test. Categorical variables are expressed as counts with percentages and were compared using Pearson’s chi-square or Fisher’s exact test. Multiple samples are compared using the non-parametric Kruskal–Wallis test. Variables with *p* values < 0.05 were considered statistically significant. The association between IBT and post-LT AKI and in-hospital mortality were evaluated using univariable and multivariable logistic regression analyses tests. Model performance was assessed in terms of both discrimination and calibration. Discrimination of the multivariable logistic regression models was evaluated using the C-index, and calibration was examined using calibration plots and the Hosmer–Lemeshow goodness-of-fit test. As this was a retrospective observational study, the sample size was determined by the number of eligible patients during the study period, and no formal *a priori* power calculation was performed. The matched cohort of 105 pairs, together with an overall incidence of post-LT AKI of 36.7% in the unmatched cohort, is expected to provide adequate power to detect clinically relevant differences in AKI. However, given the low incidence of in-hospital mortality (4.8%), the study is likely underpowered for this rare outcome, and the corresponding results should be interpreted with caution. Data were analyzed using SPSS software (version 22.0; IBM Corp.) and illustrated using GraphPad Prism 9 (GraphPad Software Inc.).

## Results

After initial screening, in total 686 patients who underwent liver transplantation were included in the analyses. Among them, 517 (75.4%) patients received an intraoperative blood transfusion (IBT group), and 169 (24.6%) patients did not (non-IBT group). The Comparisons of patients’ characteristics and operative variables between the two groups in the unmatched cohort are shown in Table 1.

Variables with significant differences (*p* < 0.05) in Table 1 were entered into univariable logistic regression analysis. Significant variables associated with IBT (*p* < 0.1) in univariable regression were entered into multivariable logistic regression analysis. Independent predictive factors of IBT for LT are shown in Supplementary Table S1. Which included sex (*p* = 0.044), weight (*p* = 0.005), liver cancer (*p* = 0.003), alcoholic hepatitis (*p* = 0.048), Child-Pugh classification (*p* = 0.006), anhepatic phase (*p* = 0.044), and intraoperative bleeding (*p* < 0.0001).

In the unmatched cohort, univariable and multivariable logistic regression analyses of IBT in relation to post-LT AKI and in-hospital mortality are presented in Supplementary Tables S2, S3, respectively. In univariable logistic regression analysis, IBT was significantly associated with an increased risk of post-LT AKI (OR: 2.993, 95% CI: 2.061–4.347, *p* < 0.0001) and in-hospital mortality (OR: 2.692, 95% CI: 1.313–5.522, *p* = 0.007). In multivariable logistic regression analyses adjusting for potential confounders, IBT remained an independent risk factor for post-LT AKI (OR: 1.741, 95% CI: 1.083–2.800, *p* = 0.022) and in-hospital mortality (OR: 1.323, 95% CI: 1.001–1.565, *p* = 0.032) in the unmatched cohort.

After propensity score matching, 105 pairs of patients with similar propensity scores were identified. Twenty preoperative and intraoperative covariates were included in the propensity score model, and the standardized mean differences (SMDs) in the unmatched and matched cohorts are presented in Supplementary Table S4. After matching, the vast majority of variables had SMD values below 0.1, indicating that covariate balance between the IBT and non-IBT groups was substantially improved. In the matched cohort, 105 patients received IBT. Among them, 17 patients (16.2%) received 2 units, 52 patients (49.5%) received 4 units, 16 patients (15.2%) received 6 units, 12 patients (11.4%) received 8 units, and 8 patients (7.6%) received 10–12 units. Thus, a total of 97 patients (92.4%) received no more than 8 units of blood.

Comparisons of patients’ characteristics and operative variables between the IBT and non-IBT groups in the matched cohort are shown in Table 2. Apart from cold ischemia time, intraoperative blood product transfusions, and intraoperative fluid balance, all other variables were not different significantly between the two groups (all *p* > 0.05).

**Table 2:** Comparisons of patients’ characteristics and operative variables between the non-intraoperative blood transfusion (non-IBT) and IBT groups in the matched cohort.

Characteristics	Total (*n* = 210)	Non-IBT (*n* = 105)	IBT (*n* = 105)	*p*-value
Demographic data				
Age (years)	54 (47,59)	55 (45,59)	53 (48,59)	0.925
Sex, male (*N*, %)	178 (84.8%)	89 (84.8%)	89 (84.8%)	1.000
Height (cm)	171 (168,175)	172 (168,175)	170 (168,175)	0.622
Weight (kg)	71 (65,80)	70 (65,80)	71 (65,79)	0.975
BMI (Kg/m^2^)	24.4 (22.3,26.8)	24.5 (22.0,26.8)	24.2 (22.5,26.8)	0.868
Primary liver disease				
Liver cancer (*N*, %)	128 (61.0%)	65 (61.9%)	63 (60.0%)	0.888
Viral B hepatitis (*N*, %)	137 (65.2%)	70 (66.7%)	67 (63.8%)	0.772
Alcoholic hepatitis (*N*, %)	26 (12.4%)	13 (12.4%)	13 (12.4%)	1.000
Autoimmune hepatitis (*N*, %)	6 (2.9%)	3 (2.9%)	3 (2.9%)	1.000
Liver cirrhosis (*N*, %)	189 (90.0%)	94 (89.5%)	95 (90.5%)	1.000
Liver failure (*N*, %)	35 (16.7%)	17 (16.2%)	18 (17.1%)	1.000
Liver complications				
Encephalopathy (*N*, %)	36 (17.1%)	17 (16.2%)	19 (18.1%)	0.855
Ascites (*N*, %)	102 (48.6%)	49 (46.7%)	53 (50.5%)	0.679
Baseline medical status				
Coronary heart diseases (*N*, %)	9 (4.3%)	4 (3.8%)	5 (4.8%)	1.000
Hypertension (*N*, %)	27 (12.9%)	14 (13.3%)	13 (12.4%)	1.000
Diabetes mellitus (*N*, %)	43 (20.5%)	21 (20.0%)	22 (21.0%)	1.000
Child-Pugh classification (*N*, %)				
A	78 (37.1%)	42 (40.0%)	36 (34.3%)	0.454
B	89 (42.4%)	40 (38.1%)	49 (46.7%)	
C	43 (20.5%)	23 (21.9%)	20 (19.0%)	
MELD score	12 (8,16)	12 (8,16)	11 (8,16)	0.824
Preoperative laboratory data				
Serum creatinine (μmol/L)	61 (52, 70)	60 (52, 68)	62 (52, 72)	0.311
AST (U/L)	44 (29, 72)	40 (29, 74)	48 (29, 71)	0.590
TB (μmol/L)	34 (18, 67)	36 (18, 66)	33 (20, 67)	0.955
Intraoperative details				
Cold ischemia time (h)	5 (5,5.4)	5 (5,6)	5 (5,5)	0.026*
Anhepatic phase (minute)	50 (44,58)	50 (43,58)	50 (45,58)	0.700
Surgical approach, classic, (*N*, %)	197 (95.6%)	101 (97.1%)	96 (94.1%)	0.293
Operative time (h)	6.30 (5.50, 7.26)	6.25 (5.51, 7.24)	6.45 (6.00, 7.20)	0.936
Intraoperative bleeding (mL)	600 (500, 900)	600 (500, 900)	700 (500, 900)	0.832
Blood product transfusions (mL)	200 (0,800)	0 (0,0)	800 (800, 1,200)	<0.0001*
Intraoperative fluid balance (mL)	3,700 (2,900, 4,250)	3,289 (2,612, 4,037)	3,940 (3,350, 4,470)	<0.0001*
Intraoperative hypotension (*N*, %)	21 (10.0%)	8 (7.6%)	13 (12.4%)	0.358
Intraoperative urine output (mL)	1,100 (825, 1,490)	1,050 (845, 1,410)	1,130 (800, 1,500)	0.599

Non-normally distributed continuous variables are displayed as a median with interquartile range (IQR) and were compared using the Mann–Whitney *U* test. Categorical variables are expressed as counts with percentages and were compared using Pearson’s chi–square or Fisher’s exact test. Multiple samples are compared using the non-parametric Kruskal–Wallis test. IBT: intraoperative blood transfusion; BMI: body mass index; MELD: model for end-stage liver disease; AST: aspartate aminotransferase; TB: serum total bilirubin. *p-value < 0.05.

**Figure 2:**
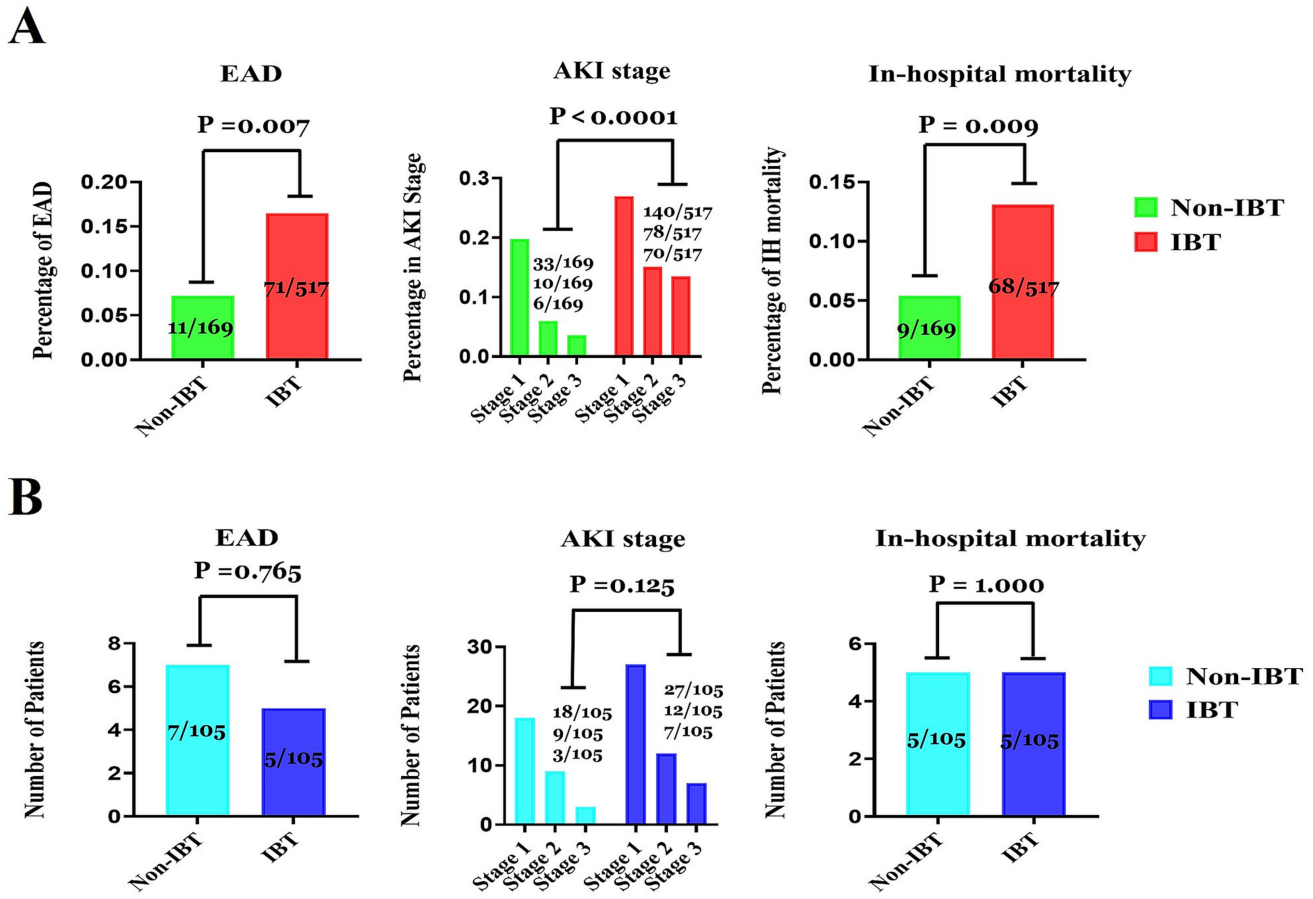
Comparisons of EAD, AKI and in-hospital mortality between the IBT and non-IBT groups in the unmatched and matched cohorts. **(A)** Unmatched Cohort; **(B)** Matched Cohort. Categorical variables were compared using Pearson’s chi–square or Fisher’s exact test. Multiple samples are compared using the non-parametric Kruskal–Wallis test. EAD: early allograft dysfunction; AKI: acute kidney injury.

Comparisons of patients’ postoperative laboratory data and clinical parameter between the IBT and non-IBT groups in the unmatched and matched cohorts are illustrated in Figure 2 and Supplementary Table S5. The ratios of EAD, post - LT AKI, re-intubation, and in-hospital mortality in the IBT group was significantly higher than in the non-IBT group in the unmatched cohort (*p* < 0.0001, *p* < 0.0001, *p* < 0.0001, and *p* = 0.009, respectively), but there was no significant difference in the ratios of EAD, post - LT AKI, re-intubation, and in-hospital mortality between these two groups in the matched cohort (*p* = 0.765, *p* = 0.125, *p* = 1.000, and *p* = 1.000, respectively; Figure 2). Furthermore, the duration of mechanical ventilation and duration of ICU stay were significantly longer in in the IBT group than in the non-IBT group in the unmatched cohort (*p* < 0.0001, and *p* < 0.0001, respectively), but not significantly different between the two groups in the matched cohort (*p* = 0.458, and *p* = 0.178, respectively; Supplementary Table S5).

In the matched cohort, univariable and multivariable logistic regression analyses of IBT in relation to post-LT AKI and in-hospital mortality are presented in Tables 3, 4, respectively. In univariable logistic regression analysis, IBT was significantly associated with increased risks of post - LT AKI (OR: 1.861, 95% CI: 1.053–3.290, *p* = 0.033) but not associated with in hospital mortality (OR: 1.000, 95% CI: 0.281–3.562, *p* = 1.000). In multivariable logistic regression analyses adjusting for potential confounders, IBT was not an independent risk factor associated with post - LT AKI (OR: 1.826, 95% CI: 0.968–3.446, *p* = 0.063) and in-hospital mortality (OR: 1.579, 95% CI: 0.251–9.935, *p* = 0.627) in the matched cohort.

**Table 3:** Univariable and multivariable logistic regression analysis for the predictors of post-LT AKI in the matched cohort.

Variables	Total	AKI	Non-AKI	UV	*p*-value	MV	*p*-value
	*N* = 210	*N* = 77	*N* = 133	OR (95% CI)		OR (95% CI)	
Age (years)	54 (47, 59)	56 (49, 62)	52 (46, 59)	1.036 (1.006–1.067)	0.019*	1.052 (1.016–1.088)	0.004*
Sex, male (*N*, %)	178 (84.8%)	59 (76.6%)	119 (89.5%)	2.593 (1.207–5.573)	0.015*	3.199 (1.287–7.949)	0.012*
Height (cm)	171 (168, 175)	170 (164, 176)	172 (169, 175)	0.972 (0.931–1.014)	0.190		
Weight (kg)	71 (65, 80)	71 (64, 79)	70 (65, 80)	1.004 (0.982–1.027)	0.710		
BMI (Kg/m^2^)	24.4 (22.3, 26.8)	24.4 (22.3, 27.0)	24.2 (22.3, 26.7)	1.045 (0.965–1.130)	0.280		
Liver cancer (*N*, %)	128 (61.0%)	36 (46.8%)	92 (69.2%)	0.391 (0.219–0.699)	0.002*		
Viral B hepatitis (*N*, %)	137 (65.2%)	44 (57.1%)	93 (69.9%)	0.573 (0.320–1.029)	0.062		
Alcoholic hepatitis (*N*, %)	26 (12.4%)	10 (13.0%)	16 (12.0%)	1.091 (0.469–2.541)	0.839		
Autoimmune hepatitis (*N*, %)	6 (2.9%)	2 (2.6%)	4 (3.0%)	0.860 (0.154–4.808)	0.864		
Liver cirrhosis (*N*, %)	189 (90.0%)	67 (87.0%)	122 (91.7%)	0.604 (0.244–1.496)	0.276		
Liver failure (*N*, %)	35 (16.7%)	18 (23.4%)	17 (12.8%)	2.082 (1.000–4.334)	0.050*		
Encephalopathy (*N*, %)	36 (17.1%)	21 (27.3%)	15 (11.3%)	2.950 (1.415–6.152)	0.004*		
Ascites (*N*, %)	102 (48.6%)	48 (62.3%)	54 (40.6%)	2.421 (1.361–4.309)	0.003*	2.069 (1.081–3.957)	0.028*
Coronary heart diseases (*N*, %)	9 (4.3%)	4 (5.2%)	5 (3.8%)	1.403 (0.365–5.389)	0.622		
Hypertension (*N*, %)	27 (12.9%)	12 (15.6%)	15 (11.3%)	1.452 (0.641–3.288)	0.371		
Diabetes mellitus (*N*, %)	43 (20.5%)	17 (22.1%)	26 (19.5%)	1.166 (0.586–2.321)	0.662		
Child-Pugh classification (*N*, %)					0.005*		
A	78 (37.1%)	18 (23.4%)	60 (45.1%)				
B	89 (42.4%)	37 (48.1%)	52 (39.1%)	2.372 (1.208–4.657)			
C	43 (20.5%)	22 (28.6%)	21 (15.8%)	3.492 (1.574–7.747)			
MELD score	12 (8, 16)	14 (10, 19)	10 (7, 14)	1.078 (1.034–1.123)	<0.0001*	1.082 (1.031–1.135)	0.001*
Preoperative Serum creatinine (μmol/L)	61 (52, 70)	57 (47, 67)	62 (55, 71)	0.985 (0.966–1.004)	0.119		
Preoperative AST (U/L)	44 (29, 72)	45 (32, 81)	42 (28, 64)	1.003 (0.999–1.006)	0.131		
Preoperative TB (μmol/L)	34 (18, 67)	52 (29, 105)	26 (16, 54)	1.002 (1.000–1.004)	0.032*		
Cold ischemia time (h)	5.0 (5.0, 5.4)	5.0 (5.0, 5.0)	5.0 (5.0, 6.0)	0.887 (0.717–1.097)	0.269		
Anhepatic phase (minute)	50 (44, 58)	49 (43, 57)	50 (44, 59)	0.991 (0.969–1.013)	0.415		
Surgical approach, classic, (*N*, %)	197 (95.6%)	75 (97.4%)	122 (94.6%)	0.465 (0.094–2.296)	0.347		
Operative time (h)	6.30 (5.50, 7.26)	6.20 (5.50, 7.14)	6.35 (5.50, 7.49)	0.904 (0.718–1.136)	0.386		
Intraoperative bleeding (mL)	600 (500, 900)	600 (500, 900)	700 (500, 900)	1.000 (1.000–1.001)	0.390		
Intraoperative fluid balance (mL)	3,700 (2,900, 4,250)	3,880 (3,112, 4,410)	3,625 (2,765, 4,180)	1.000 (1.000–1.000)	0.220		
Intraoperative hypotension (*N*, %)	21 (10.0%)	9 (11.7%)	12 (9.0%)	1.335 (0.535–3.328)	0.536		
Intraoperative urine output (mL)	1,100 (825, 1,490)	990 (770, 1,305)	1,150 (875, 1,544)	0.999 (0.998–1.000)	0.008*		
Lactic acid after surgery (mmol/L)	2.63 (1.83, 3.61)	2.91 (1.77, 3.70)	2.61 (1.83, 3.43)	1.041 (0.850–1.275)	0.699		
ALT after surgery (U/L)	360 (228, 569)	323 (199, 601)	364 (258, 548)	1.000 (1.000–1.000)	0.861		
AST after surgery (U/L)	723 (463, 1,231)	719 (444, 1,231)	724 (490, 1,239)	1.000 (1.000–1.000)	0.711		
TB after surgery (μmol/L)	58 (38, 89)	67 (49,111)	53 (32, 79)	1.004(1.000–1.007)	0.043*		
Serum creatinine after surgery (μmol/L)	63 (52,74)	67 (52, 80)	61 (54, 72)	1.019 (1.003–1.035)	0.023*	1.022 (1.003–1.042)	0.027*
EAD (*N*, %)	12 (6.5%)	8 (10.8%)	4 (3.6%)	3.273 (0.948–11.294)	0.061		
IBT (*N*, %)	105 (50%)	46 (59.7%)	59 (44.4%)	1.861 (1.053–3.290)	0.033*	1.826 (0.968–3.446)	0.063
Blood product transfusions (mL)	200 (0,800)	400 (0,800)	0 (0,800)	1.000 (1.000–1.001)	0.381		

AKI: acute kidney injury; BMI: Body mass index; MELD: Model for End-Stage Liver Disease; ALT: Alanine aminotransferase; AST: Aspartate aminotransferase; TB: Serum total bilirubin; EAD: early allograft dysfunction; IBT: intraoperative blood transfusion; RRT: renal replacement therapy; ICU: intensive care unit. *p-value < 0.05.

**Table 4:** Univariable and multivariable logistic regression analysis for the predictors of in-hospital mortality in the matched cohort.

Variables	Total	IH-mortality	Survive	UV	*p*-value	MV	*p*-value
	*N* = 210	*N* = 10	*N* = 200	OR (95% CI)		OR (95% CI)	
Age (years)	54 (47, 59)	62 (55, 64)	53 (47, 59)	1.048 (0.978–1.124)	0.185		
Sex, male (*N*, %)	178 (84.8%)	7 (70.0%)	171 (85.5%)	2.527 (0.618–10.337)	0.197		
Height (cm)	171 (168, 175)	170 (161, 172)	172 (168, 175)	0.923 (0.843–1.010)	0.080	0.884 (0.777–1.005)	0.060
Weight (kg)	71 (65, 80)	64 (56, 70)	71 (65, 80)	0.956 (0.903–1.011)	0.117		
BMI (Kg/m^2^)	24.4 (22.3, 26.8)	22.8 (19.4, 25.1)	24.4 (22.5, 26.8)	0.899 (0.745–1.085)	0.269		
Liver cancer (*N*, %)	128 (61.0%)	5 (50.0%)	123 (61.5%)	0.626 (0.175–2.233)	0.470		
Viral B hepatitis (*N*, %)	137 (65.2%)	3 (30.0%)	134 (67.0%)	0.211 (0.053–0.843)	0.028*	0.728 (0.122–4.359)	0.728
Alcoholic hepatitis (*N*, %)	26 (12.4%)	3 (30.0%)	23 (11.5%)	3.298 (0.797–13.652)	0.100		
Autoimmune hepatitis (*N*, %)	6 (2.9%)	1 (10.0%)	5 (2.5%)	4.333 (0.457–41.057)	0.201		
Liver cirrhosis (*N*, %)	189 (90.0%)	8 (80.0%)	181 (90.5%)	0.420(0.083–2.122)	0.294		
Liver failure (*N*, %)	35 (16.7%)	1 (10.0%)	34 (17%)	0.542 (0.067–4.424)	0.568		
Encephalopathy (*N*, %)	36 (17.1%)	3 (30.0%)	33 (16.5%)	2.169 (0.533–8.822)	0.279		
Ascites (*N*, %)	102 (48.6%)	7 (70.0%)	95 (47.5%)	2.579 (0.648–10.258)	0.179		
Coronary heart diseases (*N*, %)	9 (4.3%)	0 (0.0%)	9 (4.5%)	/	0.999		
Hypertension (*N*, %)	27 (12.9%)	2 (20.0%)	25 (12.5%)	1.750 (0.352–8,713)	0.494		
Diabetes mellitus (*N*, %)	43 (20.5%)	2 (20.0%)	41 (20.5%)	0.970 (0.198–4.740)	0.969		
Child-Pugh classification (*N*, %)					0.279		
A	78 (37.1%)	2 (20.0%)	76 (38.0%)				
B	89 (42.4%)	4 (40.0%)	85 (42.5%)	1.788 (0.319–10.040)			
C	43 (20.5%)	4 (40.0%)	39 (19.5%)	3.897 (0.684–22.221)			
MELD score	12 (8, 16)	14 (9, 17)	11 (8, 16)	1.046 (0.973–1.124)	0.228		
Preoperative Serum creatinine (μmol/L)	61 (52, 70)	67 (48, 72)	61 (52, 69)	1.018 (0.976–1.062)	0.402		
Preoperative AST (U/L)	44 (29, 72)	34 (25, 54)	45 (30, 73)	1.004 (1.000–1.008)	0.052		
Preoperative TB (μmol/L)	34 (18, 67)	42 (20, 77)	33 (18, 67)	1.002 (0.999–1.005)	0.173		
Cold ischemia time (h)	5.0 (5.0, 5.4)	5.0 (5.0, 5.0)	5.0 (5.0, 5.0)	1.079 (0.906–1.285)	0.393		
Anhepatic phase (minute)	50 (44, 58)	47 (44, 65)	50 (44, 58)	1.004 (0.958–1.051)	0.872		
Surgical approach, classic, (*N*, %)	197 (95.6%)	9 (90.0%)	188 (94%)	2.611 (0.294–23.186)	0.389		
Operative time (h)	6.30 (5.50, 7.26)	6.30 (6.00, 7.14)	6.30 (5.50, 7.30)	1.054 (0.640–1.736)	0.837		
Intraoperative bleeding (mL)	600 (500, 900)	850 (500, 1,000)	600 (500, 900)	1.001 (0.999–1.002)	0.453		
Intraoperative fluid balance (mL)	3,700 (2,900, 4,250)	3,685 (2,750, 4,350)	3,700 (2,900, 4,240)	1.000 (0.999–1.000)	0.935		
Intraoperative hypotension (*N*, %)	21 (10.0%)	2 (20.0%)	19 (9.5%)	2.382 (0.471–12.034)	0.294		
Intraoperative urine output (mL)	1,100 (825, 1,490)	940 (750, 1,570)	1,103 (845, 1,480)	1.000 (0.998–1.001)	0.554		
Lactic acid after surgery (mmol/L)	2.63 (1.83, 3.61)	2.92 (2.50, 3.90)	2.62 (1.83, 3.61)	1.126 (0.747–1.697)	0.570		
ALT after surgery (U/L)	360 (228, 569)	373 (260, 759)	360 (223, 562)	1.001 (1.000–1.001)	0.124		
AST after surgery (U/L)	723 (463, 1,231)	796 (343, 2,328)	719 (463, 1,231)	1.000 (1.000–1.001)	0.068		
TB after surgery (μmol/L)	58 (38, 89)	66 (45, 111)	58 (37, 89)	1.004 (0.998–1.009)	0.175		
Serum creatinine after surgery (μmol/L)	63 (52, 74)	77 (56, 106)	62 (52, 74)	1.045 (1.016–1.076)	0.003*	1.042 (1.016–1.069)	0.001*
EAD (*N*, %)	12 (6.5%)	2 (20.0%)	10 (5%)	4.771 (0.875–26.016)	0.071		
AKI stage (*N*, %)					0.130		
I	45 (21.4%)	0 (0.0%)	45 (22.5%)				
II	21 (10.0%)	3 (30.0%)	18 (9.0%)				
III	10 (4.8%)	3 (30.0%)	7 (3.5%)	2.725 (0.744–9.979)			
IBT (*N*, %)	105 (50.0%)	5 (50.0%)	100 (50%)	1.000 (0.281–3.562)	1.000	1.579 (0.251–9.935)	0.627
Blood product transfusions (mL)	200 (0,800)	400 (0,900)	200 (0,800)	1.000 (0.999–1.001)	0.981		

BMI: body mass index; MELD: model for end-stage liver disease; ALT: alanine aminotransferase; AST: aspartate aminotransferase; TB: serum total bilirubin; EAD: early allograft dysfunction; AKI: acute kidney injury; IBT: intraoperative blood transfusion; RRT: renal replacement therapy; ICU: intensive care unit. *p-value < 0.05.

Model performance was evaluated in terms of discrimination and calibration. Discrimination, assessed using the C-index, showed acceptable to good discriminative ability for post-LT AKI and in-hospital mortality in both the unmatched and matched cohorts. Calibration was assessed using calibration plots (Supplementary Figure S1) and the Hosmer–Lemeshow goodness-of-fit test, which indicated adequate fit for all models. These results suggest that the regression models adequately fulfilled the assumptions of the analyses performed.

## Discussion

Many studies have reported that perioperative transfusion is associated with severe postoperative complications and increased short-term and long-term mortality rates. However, the grouping criteria in these studies often involve a large amount of transfusion, and the time frame for calculating transfusion volume is often limited to 48 h after surgery.

In this retrospective study conducted at our center, we aimed to investigate the impact of intraoperative transfusion on postoperative AKI and in-hospital mortality rate. Our study found no correlation between intraoperative transfusion and postoperative AKI or in-hospital mortality rate after propensity score matching for patient gender, BMI, underlying disease, and intraoperative blood loss.

Due to advancements in anesthesia management and surgical techniques, the amount of blood loss and demand for blood products during the LT procedure has significantly decreased in the past decade (5, 13). Autologous transfusion during liver transplantation can greatly reduce the need for allogeneic transfusion. However, significant intraoperative blood loss and allogeneic transfusion during surgery remain common. In our study, 75% of patients received allogeneic transfusions during surgery. Our center is one of the largest liver transplantation centers in Beijing, completing over 200 liver transplant surgeries annually and accumulating rich experience in surgery and clinical care. Compared to previous studies, our study has a relatively larger sample size, with 793 patients included in the selection process and 686 patients included in the data analysis. In one study, 227 patients were included. After propensity score matching, 59 patients were assigned to the massive transfusion (MT) group, and 59 patients were assigned to the non-MT group (13). Our study used strict matching criteria, resulting in 105 pairs of patients being included in the analysis, which increases accuracy and model persuasiveness.

When analyzing all patients in this cohort, intraoperative blood transfusion in liver transplant patients was significantly associated with postoperative EAD, AKI, and in-hospital mortality. However, due to the many confounding factors between the IBT group and non-IBT group, these factors could lead to both poor prognosis and blood transfusion, so the study results should be interpreted with caution, as demonstrated in this study, the relationship between intraoperative blood transfusion and poor patient prognosis may simply be coincidental. Using the propensity score matching method to match patients’ baseline data and intraoperative factors simulated a randomized controlled study (37, 38). After adjusting with univariable and multivariable regression analysis, the study results suggest that IBT is no longer associated with postoperative EAD, AKI, and hospital mortality. Therefore, for liver transplant patients, the relationship between intraoperative blood transfusion and prognosis is due to patients’ baseline data and intraoperative factors rather than the blood transfusion itself.

Similar conclusions have been drawn in studies on the correlation between blood transfusion and the prognosis of other types of surgical patients (39–41). In a large-scale clinical study of liver resection in patients with liver cancer, prior to propensity score matching, Perioperative blood transfusion (PBT) was associated with an increased risk of overall survival (OS) and recurrence-free survival (RFS), but after propensity score matching, PBT was no longer associated with an increased risk of OS and RFS (42).

There are several differences between our study and previous studies, which is why our study has different results. We had more patients in our study, so we were able to use more rigorous propensity score matching criteria. Disease severity and intraoperative blood loss can both affect intraoperative transfusion. In addition, our preliminary studies found that intraoperative hypotension were related to postoperative AKI (43), and we included all these factors in our propensity score matching. Our study focused primarily on intraoperative transfusions, while other studies set the bleeding calculation time at 48 h after surgery, which may include the influence of other postoperative factors on prognosis, such as continuous bleeding from the surgical incision. This is directly related to prognosis. A newer study found that fresh red blood cell transfusions during surgery were related to patient prognosis (22), but our study did not differentiate between the freshness of the blood.

The most important factor is the amount of blood transfusion. Many studies use a cutoff of massive transfusion, which is six or even 10 units of blood (13, 16). In our study we strictly matched the IBT and non-IBT groups. Patients with lower blood transfusion volume were selected from the IBT group to match with the non-IBT group. After propensity score matching, 105 pairs of patients were obtained. Among the 105 patients who received IBT, the vast majority, 97 patients (92.4%), received no more than 8 units of blood. We attempted to match the non-IBT group with patients who received more than eight units of blood transfusion. We performed propensity score matching with the 20 covariables mentioned earlier, but we were unable to find any cases that matched. We even relaxed the caliper value to 0.2, but we still could not find a match. This further indicates that factors such as disease severity and surgical factors affect intraoperative transfusion and are related to prognosis. In summary, our study at least indicates that a relatively small amount of blood transfusion itself does not affect patient prognosis. This provides some reference value for the intraoperative blood transfusion strategy for liver transplant patients.

Our study has some limitations. First, due to the hospital’s requirements for patient privacy and ethics, factors related to the donor were not included in the analysis. Second, this was a retrospective study rather than a randomized controlled study, but given the uniqueness of this issue, it is almost impossible to conduct a randomized controlled study. Our study used propensity score matching and subsequent multivariable retrospective analysis to simulate a controlled study as much as possible. Third, because Hgb, PLT, and INR were measured at multiple, non-standardized time points and were not comparable across patients, we were unable to include these variables in our analysis. Future prospective studies with predefined laboratory time points are needed to better assess their impact on transfusion practice. Fourth, the follow-up period was relatively short and detailed information on perioperative and postoperative coagulation status and bleeding was limited. Therefore, we cannot exclude the possibility that the impact of intraoperative transfusion on AKI may become more evident over longer-term follow-up, and further prospective studies with extended follow-up are warranted to clarify this relationship. Fifth, this was a single-center study. While this provides reasonable power for evaluating relatively frequent outcomes such as post-LT AKI, the low incidence of in-hospital mortality (4.8%) means that the study is likely underpowered for this rare endpoint, and these results should therefore be interpreted with caution. Further confirmation in multicenter studies, both domestically and internationally, is warranted. Sixth, it should be noted that although our study found that limited intraoperative blood transfusion itself was not related to postoperative AKI and hospital mortality in liver transplant patients, blood transfusions can not only increase hospital costs but also lead to transfusion reactions and ABO incompatibility issues. Lastly, in our matched cohort, the vast majority of patients, 97 (92.4%), received no more than 8 units of blood; therefore, our findings may not be fully applicable to cases involving massive transfusion. This suggests that patients requiring more than 8 units of transfusion may have a more severe condition than those who do not require transfusion. Nevertheless, this study demonstrates that after rigorous matching for factors such as disease severity, blood transfusions themselves do not affect patient outcomes.

## Conclusion

Using propensity score matching and multivariable logistic regression analysis, it was found that there was no statistically significant correlation between intraoperative allogeneic blood transfusion and postoperative AKI and in-hospital mortality. In our matched cohort, the vast majority of patients, received no more than 8 units of blood. The relationship between intraoperative blood transfusion and poor prognosis is due to the requirement for blood transfusion in the context of surgical factors, rather than the intraoperative blood transfusion itself. Multicenter, prospective studies are needed in the future to validate our findings.

## Data Availability

The datasets presented in this article are not readily available because of the need to protect patient privacy and in accordance with hospital policies. Requests to access the datasets should be directed to Zhipeng Wu at 13141404452@163.com.

## Glossary

IBT: intraoperative blood transfusion
AKI: acute kidney injury
LT: liver transplant
IH mortality: In-Hospital Mortality
PSM: propensity score matching
OR: odds ratio
95% CI: 95% confidence interval
EAD: early allograft dysfunction
RCTs: randomized controlled trials
CT: computed Tomography
BMI: body mass index
MELD: model for end-stage liver disease
ALT: alanine aminotransferase
AST: aspartate aminotransferase
TB: serum total bilirubin
RRT: renal replacement therapy
ICU: intensive care unit
KDIGO: 2012 kidney disease improving global outcomes
SMD: standardized mean difference
SD: standard deviation
IQR: interquartile range
ABO: ABO blood group system
PBT: perioperative blood transfusion
OS: overall survival
RFS: recurrence-free survival.
