# Supplementary material for: Intraoperative allogeneic blood transfusion is not associated with postoperative acute kidney injury and in-hospital mortality in liver transplantation patients: a propensity score matching analysis
Source: Front Med (Lausanne). 2026 Jun 23;13:1748464. doi: 10.3389/fmed.2026.1748464 (PMC13338835; doi:10.3389/fmed.2026.1748464)
Supplement: Supplementary file 1 [file Data_Sheet_1.DOCX]

**Additional file 1**

**Table S1. Univariable and multivariable logistic regression analyses for predictors of intraoperative blood transfusion (IBT) in the unmatched cohort.**

| Variables | UV |  |  |  | MV |  |  |
| --- | --- | --- | --- | --- | --- | --- | --- |
|  | Wald | OR (95% CI) | P-Value |  | Wald | OR (95% CI) | P-Value |
| Sex, male | 6.86 | 1.957 (1.184-3.234) | 0.009* |  | 4.056 | 2.189 (1.060-4.520) | 0.044* |
| Height | 0.687 | 1 (0.998-1.002) | 0.687 |  |  |  |  |
| Weight | 4.982 | 0.985 (0.972-0.998) | 0.026* |  | 7.899 | 0.969 (0.949-0.989) | 0.005* |
| Liver cancer | 51.95 | 0.238 (0.161-0.351) | ＜0.0001* |  | 8.790 | 0.469 (0.263-0.838) | 0.003* |
| Viral B hepatitis | 7.243 | 0.607 (0.422-0.873) | 0.007* |  |  |  |  |
| Alcoholic hepatitis | 9.428 | 2.346 (1.361-4.042) | 0.002* |  | 3.907 | 2.289 (1.096-4.781) | 0.048* |
| Autoimmune hepatitis | 3.831 | 2.857 (0.999-8.173) | 0.050* |  |  |  |  |
| Liver failure | 40.147 | 5.358 (3.188-9.006) | ＜0.0001* |  | 2.794 | 2.775 (1.212-6.354) | 0.095 |
| Encephalopathy (N,%) | 13.551 | 2.753 (1.606-4.720) | ＜0.0001* |  |  |  |  |
| Ascites (N,%) | 31.898 | 2.816 (1.966-4.034) | ＜0.0001* |  |  |  |  |
| Child-Pugh classification |  |  | ＜0.0001* |  | 7.638 | 2.605 (1.518-4.468) | 0.006* |
| A |  |  |  |  |  |  |  |
| B | 41.391 | 3.963 (2.605-6.028) |  |  |  |  |  |
| C | 70.926 | 9.282 (5.527-15.590) |  |  |  |  |  |
| MELD score | 41.595 | 1.088 (1.060-1.116) | ＜0.0001* |  |  |  |  |
| Serum creatinine | 3.501 | 0.991 (0.982-1.000) | 0.061 |  |  |  |  |
| AST | 0.676 | 1.001 (0.999-1.002) | 0.411 |  |  |  |  |
| TB | 30.203 | 1.005 (1.003-1.007) | ＜0.0001* |  |  |  |  |
| Anhepatic phase | 21.426 | 1.035 (1.020-1.051) | ＜0.0001* |  | 4.057 | 1.017 (0.997-1.037) | 0.044* |
| Surgical approach, classic | 12.948 | 4.292 (1.941-9.489) | ＜0.0001* |  |  |  |  |
| Operative time | 29.202 | 1.495 (1.292-1.730) | ＜0.0001* |  |  |  |  |
| Intraoperative bleeding | 80.184 | 1.003 (1.002-1.003) | ＜0.0001* |  | 61.122 | 1.003 (1.002-1.004) | ＜0.0001* |
| Intraoperative hypotension | 15.840 | 2.849 (1.701-4.772) | ＜0.0001* |  |  |  |  |

Abbreviations: IBT: intraoperative blood transfusion; MELD: Model for End-Stage Liver Disease; AST: Aspartate aminotransferase; TB: Serum total bilirubin.

*p-value <0.05.

**Table S2. Univariable and multivariable logistic regression analysis for the predictors of post-LT AKI in the unmatched cohort.**

| Variables | Total | AKI | Non-AKI | UV |  |  | MV |  |
| --- | --- | --- | --- | --- | --- | --- | --- | --- |
|  | N=686 | N=338 | N=348 | OR (95% CI) | P-Value |  | OR (95% CI) | P-Value |
| Age (years) | 53 (45,59) | 55 (46,61) | 52 (44,59) | 1.022 (1.007-1.037) | 0.004* |  | 1.022 (1.003-1.041) | 0.025* |
| Sex, male (N,%) | 551 (80.3%) | 254 (75.1%) | 297 (85.3%) | 1.926 (1.309-2.834) | 0.001* |  | 2.077 (1.267-3.406) | 0.004* |
| Height (cm) | 170 (166,175) | 170 (165,175) | 172 (168,175) | 1.001 (0.999-1.002) | 0.520 |  |  |  |
| Weight (kg) | 70 (63,78) | 70 (62,78) | 70 (64,79) | 0.997 (0.986-1.009) | 0.631 |  |  |  |
| BMI (Kg/m**^2^**) | 24.2 (22.0,26.3) | 24.2 (22.0,26.3) | 24.1 (22.1,26.3) | 1.020 (0.980-1.062) | 0.323 |  |  |  |
| Liver cancer (N,%) | 340 (49.6%) | 138 (40.8%) | 202 (58.0%) | 0.499 (0.368-0.676) | ＜0.0001* |  | 0.643 (0.472-0.969) | 0.035* |
| Viral B hepatitis (N,%) | 390 (56.9%) | 182 (53.8%) | 208 (59.8%) | 0.785 (0.580-1.063) | 0.118 |  |  |  |
| Alcoholic hepatitis (N,%) | 126 (18.4%) | 72 (21.3%) | 54 (15.5%) | 1.474 (0.998-2.176) | 0.051 |  |  |  |
| Autoimmune hepatitis (N,%) | 38 (5.5%) | 24 (7.1%) | 14 (4.0%) | 1.823 (0.927-3.588) | 0.082 |  |  |  |
| Liver cirrhosis (N,%) | 612 (89.2%) | 305 (90.2%) | 307 (88.2%) | 1.234 (0.760-2.005) | 0.395 |  |  |  |
| Liver failure (N,%) | 223 (32.5%) | 133 (39.3%) | 88 (25.3%) | 1.828 (1.332-2.507) | ＜0.0001* |  |  |  |
| Encephalopathy (N,%) | 141 (20.6%) | 86 (25.4%) | 55 (15.8%) | 1.639 (1.138-2.361) | 0.008* |  |  |  |
| Ascites (N,%) | 397 (58%) | 219 (64.8%) | 178 (51.4%) | 1.737 (1.278-2.361) | ＜0.0001* |  |  |  |
| Coronary heart diseases (N,%) | 12 (1.7%) | 5 (1.5%) | 7 (2%) | 0.731 (0.230-2.328) | 0.596 |  |  |  |
| Hypertension (N,%) | 82 (12.0%) | 47 (13.9%) | 35 (10.1%) | 1.444 (0.907-2.301) | 0.122 |  |  |  |
| Diabetes mellitus (N,%) | 118 (17.2%) | 57 (16.9%) | 61 (17.5%) | 0.954 (0.642-1.419) | 0.818 |  |  |  |
| Child-Pugh classification (N,%) |  |  |  |  | ＜0.0001* |  |  |  |
| A | 170 (24.7%) | 59 (17.5%) | 110 (31.9%) |  |  |  |  |  |
| B | 276 (40.3%) | 130 (38.5%) | 145 (42.0%) | 1.672 (1.126-2.481) |  |  |  |  |
| C | 240 (35.0%) | 149 (44.1%) | 90 (26.1%) | 3.087 (2.047-4.653) |  |  |  |  |
| MELD score | 14 (9,21) | 16 (11,24) | 11 (8,17) | 1.051 (1.033-1.070) | ＜0.0001* |  |  |  |
| Preoperative Serum creatinine (μmol/L) | 59 (48,70) | 56 (44,69) | 60 (51,70) | 0.987 (0.979-0.996) | 0.004* |  |  |  |
| Preoperative AST (U/L) | 47 (32,80) | 51 (33,84) | 45 (30,77) | 1.001 (1.000-1.002) | 0.215 |  |  |  |
| Preoperative TB (μmol/L) | 53 (25,194) | 76 (36,268) | 37 (20,105) | 1.002 (1.001-1.002) | ＜0.0001* |  |  |  |
| Cold ischemia time (h) | 5.0 (5.0,6.0) | 5.0 (5.0,5.5) | 5.0 (5.0,6.0) | 0.975 (0.897-1.059) | 0.545 |  |  |  |
| Anhepatic phase (minute) | 52 (46,65) | 53 (46,65) | 51 (45,63) | 1.007 (0.998-1.015) | 0.109 |  |  |  |
| Surgical approach, classic, (N,%) | 584 (86.9%) | 290 (86.3%) | 294 (87.5%) | 1.110 (0.709-1.739) | 0.647 |  |  |  |
| Operative time (h) | 7 (6.09,7.75) | 7.01 (6.11,8.00) | 6.56 (6.04,7.63) | 1.115 (1.008-1.233) | 0.034* |  |  |  |
| Intraoperative bleeding (ml) | 1000 (600,1600) | 1000 (700,2000) | 800 (500,1300) | 1.000 (1.000-1.000) | 0.001* |  | 1.000 (1.000-1.000) | 0.029* |
| Intraoperative fluid balance (ml) | 4022 (3100,5000) | 4310 (3500,5275) | 3800 (2890,4600) | 1.000 (1.000-1.000) | ＜0.0001* |  | 1.000 (1.000-1.000) | 0.094 |
| Intraoperative hypotension (N,%) | 158 (23.1%) | 98 (29.1%) | 60 (17.2%) | 1.960 (1.362-2.821) | ＜0.0001* |  |  |  |
| Intraoperative urine output (ml) | 1090 (800,1400) | 1000 (740,1300) | 1130 (900,1510) | 0.999 (0.999-0.999) | ＜0.0001* |  | 1.000 (0.999-1.000) | 0.038* |
| Lactic acid after surgery (mmol/L) | 2.83 (1.93,4.14) | 3.08 (1.90,4.42) | 2.67 (1.94,3.64) | 1.114 (1.030-1.205) | 0.007* |  |  |  |
| ALT after surgery (U/L) | 408 (249,695) | 437 (242,794) | 380 (257,624) | 1.000 (1.000-1.000) | 0.004* |  |  |  |
| AST after surgery (U/L) | 856 (511,1492) | 934 (517,1848) | 797 (506,624) | 1.000 (1.000-1.000) | ＜0.0001* |  |  |  |
| TB after surgery (μmol/L) | 74 (47,136) | 92 (54,166) | 64 (40,109) | 1.003 (1.001-1.005) | ＜0.0001* |  |  |  |
| Serum creatinine after surgery (μmol/L) | 64 (48,79) | 66 (52,81) | 59 (49,70) | 1.019 (1.011-1.027) | ＜0.0001* |  | 1.017 (1.006-1.027) | ＜0.0001* |
| EAD (N,%) | 82 (14.1%) | 59 (19.9%) | 23 (8%) | 2.847 (1.705-2.847) | ＜0.0001* |  | 2.149 (1.204-3.838) | 0.010* |
| IBT (N,%) | 517 (75.4%) | 288 (85.2%) | 229 (65.8%) | 2.993 (2.061-4.347) | ＜0.0001* |  | 1.741 (1.083-2.800) | 0.022* |
| Blood product transfusions (ml) | 800 (400,1600) | 1200 (800,2000) | 800 (0,1600) | 1.000 (1.000-1.000) | ＜0.0001* |  |  |  |

Abbreviations: AKI: acute kidney injury; BMI: Body mass index; MELD: Model for End-Stage Liver Disease; ALT: Alanine aminotransferase; AST: Aspartate aminotransferase; TB: Serum total bilirubin; EAD: early allograft dysfunction; IBT: intraoperative blood transfusion; RRT: renal replacement therapy; ICU: Intensive Care Unit.

*p-value <0.05.

**Table S3. Univariable and multivariable logistic regression analysis for the predictors of in-hospital mortality in the unmatched cohort.**

| Variables | Total | IH-mortality | Survive | UV |  |  | MV |  |
| --- | --- | --- | --- | --- | --- | --- | --- | --- |
|  | N=686 | N=77 | N=609 | OR (95% CI) | P-Value |  | OR (95% CI) | P-Value |
| Age (years) | 53 (45,59) | 58 (53,64) | 53 (45,59) | 1.048 (1.022-1.075) | ＜0.0001* |  | 1.052 (1.010-1.096) | 0.014* |
| Sex, male (N,%) | 551 (80.3%) | 49 (63.6%) | 502 (82.4%) | 2.681 (1.611-4.460) | ＜0.0001* |  |  |  |
| Height (cm) | 170 (166,175) | 170 (160,174) | 170 (167,175) | 1.001 (0.999-1.003) | 0.344 |  |  |  |
| Weight (kg) | 70 (63,78) | 67 (60,75) | 70 (64,79) | 0.975 (0.957-0.994) | 0.009* |  | 0.960 (0.930-0.990) | 0.010* |
| BMI (Kg/m**^2^**) | 24.2 (22.0,26.3) | 24.0 (21.1,26.0) | 24.2 (22.0,26.3) | 0.990 (0.929-1.056) | 0.768 |  |  |  |
| liver cancer (N,%) | 340 (49.6%) | 33 (42.9%) | 307 (50.4%) | 0.738 (0.457-1.191) | 0.213 |  |  |  |
| Viral B hepatitis (N,%) | 390 (56.9%) | 39 (50.6%) | 351 (57.6%) | 0.754 (0.469-1.213) | 0.245 |  |  |  |
| Alcoholic hepatitis (N,%) | 126 (18.4%) | 14 (18.2%) | 112 (18.4%) | 0.986 (0.533-1.823) | 0.964 |  |  |  |
| Autoimmune hepatitis (N,%) | 38 (5.5%) | 6 (7.8%) | 32 (5.3%) | 1.524 (0.616-3.771) | 0.362 |  |  |  |
| Liver cirrhosis (N,%) | 612 (89.2%) | 68 (88.3%) | 544 (89.3%) | 0.903 (0.430-1.895) | 0.787 |  |  |  |
| Liver failure (N,%) | 223 (32.5%) | 26 (33.8%) | 197 (32.3%) | 1.032 (0.637-1.671) | 0.898 |  |  |  |
| Encephalopathy (N,%) | 141 (20.6%) | 23 (29.9%) | 118 (19.4%) | 1.592 (0.985-2.574) | 0.058 |  |  |  |
| Ascites (N,%) | 397 (58%) | 44 (57.1%) | 353 (58.2%) | 0.959 (0.594-1.549) | 0.865 |  |  |  |
| Coronary heart diseases (N,%) | 12 (1.7%) | 1 (1.3%) | 11 (1.8%) | 0.715 (0.091-5.618) | 0.750 |  |  |  |
| Hypertension (N,%) | 82 (12.0%) | 13 (16.9%) | 69 (11.3%) | 1.590 (0.833-3.035) | 0.160 |  |  |  |
| Diabetes mellitus (N,%) | 118 (17.2%) | 11 (14.3%) | 107 (17.6%) | 0.782 (0.400-1.530) | 0.473 |  |  |  |
| Child-Pugh classification (N,%) |  |  |  |  |  |  |  |  |
| A | 170 (24.7%) | 13 (16.9%) | 156 (25.7%) |  | 0.086 |  |  |  |
| B | 276 (40.3%) | 29 (37.7%) | 246 (40.6%) | 1.415 (0.714-2.804) |  |  |  |  |
| C | 240 (35.0%) | 35 (45.5%) | 204 (33.7%) | 2.059 (1.054-4.023) |  |  |  |  |
| MELD score | 14(9,21) | 15 (9,22) | 13 (9,21) | 1.008 (0.983-1.033) | 0.538 |  |  |  |
| Preoperative Serum creatinine (μmol/L) | 59 (48,70) | 58 (47,70) | 59 (48,70) | 0.999 (0.986-1.012) | 0.881 |  |  |  |
| Preoperative AST (U/L) | 47 (32,80) | 51 (36,99) | 47 (32,79) | 1.001 (1.000-1.002) | 0.097 |  |  |  |
| Preoperative TB (μmol/L) | 53 (25,194) | 92 (36,388) | 51 (24,181) | 1.001 (1.000-1.003) | 0.006* |  |  |  |
| Cold ischemia time (h) | 5.0 (5.0,6.0) | 5.0 (5.0,8.0) | 5 (5.0,5.5) | 1.128 (1.014-1.255) | 0.027* |  |  |  |
| Anhepatic phase (minute) | 52 (46,65) | 60 (50,76) | 52 (45,62) | 1.020 (1.010-1.031) | ＜0.0001* |  |  |  |
| Surgical approach, classic, (N,%) | 584 (86.9%) | 55 (71.4%) | 529 (88.9%) | 3.206 (1.837-5.594) | ＜0.0001* |  |  |  |
| Operative time (h) | 7.00 (6.09,7.75) | 7.50 (6.50,9.00) | 6.55 (6.06,7.55) | 1.466 (1.273-1.688) | ＜0.0001* |  |  |  |
| Intraoperative bleeding (ml) | 1000 (600,1600) | 1700 (1000,3500) | 900 (600,1500) | 1.000 (1.000-1.000) | ＜0.0001* |  | 1.000 (0.999-1.000) | 0.059 |
| Intraoperative fluid balance (ml) | 4022 (3100,5000) | 4781 (3500,6220) | 3958 (3050,4900) | 1.000 (1.000-1.000) | ＜0.0001* |  |  |  |
| Intraoperative hypotension (N,%) | 158 (23.1%) | 34 (44.2%) | 124 (20.4%) | 3.093 (1.892-5.054) | ＜0.0001* |  |  |  |
| Intraoperative urine output (ml) | 1090 (800,1400) | 943 (655,1380) | 1100 (820,1400) | 0.999 (0.999-0.999) | 0.005* |  |  |  |
| Lactic acid after surgery (mmol/L) | 2.83 (1.93,4.14) | 4.57 (2.52,6.99) | 2.76 (1.90,3.97) | 1.411 (1.264-1.576) | ＜0.0001* |  | 1.332 (1.163-1.525) | ＜0.0001* |
| ALT after surgery (U/L) | 408 (249,695) | 699 (291,1270) | 390 (237,641) | 1.001 (1.000-1.001) | ＜0.0001* |  |  |  |
| AST after surgery (U/L) | 856 (511,1492) | 1324 (629,2858) | 838 (502,1400) | 1.000 (1.000-1.000) | ＜0.0001* |  |  |  |
| TB after surgery (μmol/L) | 74 (47,136) | 96 (57,180) | 72 (47,131) | 1.002 (1.001-1.004) | 0.091 |  |  |  |
| Serum creatinine after surgery (μmol/L) | 64 (48,79) | 72 (54,90) | 61 (50,74) | 1.021 (1.011-1.030) | ＜0.0001* |  |  |  |
| EAD (N,%) | 82 (14.1%) | 22 (47.8%) | 60 (11.2%) | 7.272 (3.843-13.761) | ＜0.0001* |  | 3.715 (1.608-8.580) | 0.002* |
| AKI stage (N,%) |  |  |  |  | ＜0.0001* |  | 2.138 (1.520-3.007) | ＜0.0001* |
| I | 174 (25.3%) | 11 (14.3%) | 162 (26.6%) | 1.114 (0.521-2.380) |  |  |  |  |
| II | 88 (12.8%) | 15 (19.5%) | 73 (12.0%) | 3.370 (1.647-6.895) |  |  |  |  |
| III | 76 (11.1%) | 31 (40.3%) | 45 (7.4%) | 11.298 (5.941-21.484) |  |  |  |  |
| IBT (N,%) | 517 (75.4%) | 68 (88.3%) | 449 (73.7%) | 2.692 (1.313-5.522) | 0.007* |  | 1.313 (1.101-1.565) | 0.038* |
| Blood product transfusions (ml) | 800 (400,1600) | 2000 (800,2400) | 800 (0,1600) | 1.001 (1.001-1.001) | ＜0.0001* |  |  |  |

Abbreviations: BMI: Body mass index; MELD: Model for End-Stage Liver Disease; ALT: Alanine aminotransferase; AST: Aspartate aminotransferase; TB: Serum total bilirubin; EAD: early allograft dysfunction; AKI: acute kidney injury; IBT: intraoperative blood transfusion; RRT: renal replacement therapy; ICU: Intensive Care Unit.

*p-value <0.05.

**Table S4. Baseline characteristics and operative variables of patients in the unmatched and matched cohorts.**

| Variables | Unmatched Cohort (n=686) | | |  | Matched Cohort (n=210) | | |
| --- | --- | --- | --- | --- | --- | --- | --- |
|  | Non-IBT (n=169) | IBT (n=517) | SMD |  | Non-IBT (n=105) | IBT (n=105) | SMD |
| Demographic data |  |  |  |  |  |  |  |
| Sex, male (N,%) | 148 (87.6%) | 403 (77.9%) | 0.25 |  | 89 (84.8%) | 89 (84.8%) | 0.00 |
| BMI (kg/m²), mean ± SD | 24.67 ± 3.55 | 24.30 ± 3.86 | 0.10 |  | 24.46 ± 3.61 | 24.65 ± 3.55 | 0.05 |
| Primary liver disease |  |  |  |  |  |  |  |
| Liver cancer (N,%) | 126 (74.6%) | 214 (41.4%) | 0.71 |  | 65 (61.9%) | 63 (60.0%) | 0.04 |
| Viral B hepatitis (N,%) | 110 (65.1%) | 280 (54.2%) | 0.22 |  | 70 (66.7%) | 67 (63.8%) | 0.06 |
| Alcoholic hepatitis (N,%) | 18 (10.7%) | 108 (20.9%) | 0.28 |  | 13 (12.4%) | 13 (12.4%) | 0.00 |
| Autoimmune hepatitis (N,%) | 4 (2.4%) | 34 (6.6%) | 0.20 |  | 3 (2.9%) | 3 (2.9%) | 0.00 |
| Liver cirrhosis (N,%) | 146 (86.4%) | 466 (90.1%) | 0.12 |  | 94 (89.5%) | 95 (90.5%) | 0.03 |
| Liver failure (N,%) | 19 (11.2%) | 202 (39.1%) | 0.73 |  | 17 (16.2%) | 18 (17.1%) | 0.02 |
| Liver complications |  |  |  |  |  |  |  |
| Encephalopathy (N,%) | 18 (10.7%) | 123 (23.8%) | 0.34 |  | 17 (16.2%) | 19 (18.1%) | 0.05 |
| Ascites (N,%) | 66 (39.1%) | 331 (64.3%) | 0.51 |  | 49 (46.7%) | 53 (50.5%) | 0.08 |
| Baseline medical status |  |  |  |  |  |  |  |
| Coronary heart diseases (N,%) | 5 (3.0%) | 7 (1.4%) | 0.13 |  | 4 (3.8%) | 5 (4.8%) | 0.05 |
| Hypertension (N,%) | 26 (15.4%) | 56 (10.8%) | 0.13 |  | 14 (13.3%) | 13 (12.4%) | 0.03 |
| Diabetes mellitus (N,%) | 37 (21.9%) | 81 (15.7%) | 0.15 |  | 21 (20.0%) | 22 (21.0%) | 0.02 |
| Child-Pugh classification (N,%) |  |  |  |  |  |  |  |
| A | 86 (50.9%) | 83 (16.1%) | 0.78 |  | 42 (40.0%) | 36 (34.3%) | 0.12 |
| B | 58 (34.3%) | 217 (42.2%) | 0.16 |  | 40 (38.1%) | 49 (46.7%) | 0.18 |
| C | 25 (14.8%) | 214 (41.6%) | 0.66 |  | 23 (21.9%) | 20 (19.0%) | 0.07 |
| MELD score, mean ± SD | 11.7 ± 6.6 | 17.0 ± 9.6 | 0.59 |  | 12 (8,16) | 11 (8,16) | 0.05 |
| Preoperative laboratory data |  |  |  |  |  |  |  |
| Serum creatinine (μmol/L), mean ± SD | 62.59 ± 15.01 | 59.58 ± 19.00 | 0.18 |  | 60.23 ± 13.99 | 62.91 ± 15.54 | 0.18 |
| TB (μmol/L), mean ± SD | 70.8 ± 124.4 | 173.2 ± 204.7 | 0.61 |  | 87.5 ± 147.7 | 96.4 ± 163.6 | 0.06 |
| Operative data |  |  |  |  |  |  |  |
| Operative time (h), mean ± SD | 6.47 ± 1.26 | 7.22 ± 1.56 | 0.52 |  | 6.58 ± 1.34 | 6.52 ± 1.16 | 0.05 |
| Intraoperative bleeding (ml), mean ± SD | 626.0 ± 296.0 | 1732.0 ± 2407.0 | 0.60 |  | 715 ± 324 | 728 ± 353 | 0.04 |
| Intraoperative hypotension, n (%) | 19 (11.2%) | 139 (26.9%) | 0.39 |  | 8 (7.6%) | 13 (12.4%) | 0.16 |

Abbreviations: IBT, intraoperative blood transfusion; SMD, standardized mean difference; SD, standard deviation; BMI, body mass index; MELD, Model for End-Stage Liver Disease; TB, serum total bilirubin. *p-value < 0.05.

**Table S5. Comparisons of postoperative laboratory data and clinical parameters between the non-intraoperative blood transfusion (non‑IBT) and IBT groups in the unmatched and matched cohorts.**

| **Variable** | **Unmatched Cohort** | | | | **Matched Cohort** | | | |
| --- | --- | --- | --- | --- | --- | --- | --- | --- |
|  | **Total (n=686)** | **Non-IBT (n=169)** | **IBT (n=517)** | ***P-Value*** | **Total (n=210)** | **Non-IBT (n=105)** | **IBT (n=105)** | ***P-Value*** |
| **Postoperative laboratory data** |  |  |  |  |  |  |  |  |
| Lactic acid (mmol/L) | 2.83 (1.93,4.14) | 2.73 (1.86,3.91) | 2.89 (1.97,4.33) | 0.091 | 2.63 (1.83,3.61) | 2.53 (1.79,3.70) | 2.68 (1.93,3.69) | 0.181 |
| ALT (U/L) | 408 (249,695) | 382 (247,637) | 413 (249,725) | 0.319 | 360 (228,569) | 370 (258,656) | 351 (209,521) | 0.391 |
| AST (U/L) | 856 (511,1492) | 747 496,1231) | 897 (514,1572) | 0.016* | 723 (463,1231) | 750 (524,1275) | 690 (447,1211) | 0.411 |
| TB (μmol/L) | 74 (47,136) | 49 (32,72) | 89 (55,165) | ＜0.0001* | 58 (38,89) | 56 (38,85) | 58 (39,100) | 0.595 |
| Serum creatinine (μmol/L) | 64 (48,79) | 62 (55,75) | 61 (48,69) | 0.616 | 63 (52,74) | 60 (52,70) | 67 (53,77) | 0.057 |
| **Postoperative clinical parameter** |  |  |  |  |  |  |  |  |
| EAD (N,%) | 82 (14.1%) | 11 (7.1%) | 71 (16.6%) | 0.007* | 12 (6.5%) | 7 (7.5%) | 5 (5.4%) | 0.765 |
| AKI stage (N,%) |  |  |  | ＜0.0001* |  |  |  | 0.125 |
| I | 174 (25.3%) | 33 (19.5%) | 140 (27.1%) |  | 45 (21.4%) | 18 (17.1%) | 27 (25.7%) |  |
| II | 88 (12.8%) | 10 (5.9%) | 78 (15.1%) |  | 21 (10.0%) | 9 (8.6%) | 12 (11.4%) |  |
| III | 76 (11.1%) | 6 (3.6%) | 70 (13.6%) |  | 10 (4.8%) | 3 (2.9%) | 7 (6.7%) |  |
| RRT (N,%) | 28 (4.9%) | 3 (2.1%) | 25 (5.9%) | 0.111 | 4 (2.3%) | 1 (1.2%) | 3 (3.4%) | 0.334 |
| Duration of mechanical ventilation (h) | 22 (17,47) | 19 (16,24) | 24 (17,52) | ＜0.0001* | 19 (16,27) | 20 (16,29) | 19 (14,25) | 0.458 |
| Re-intubation (N,%) | 54 (9.9%) | 8 (5.6%) | 46 (11.4%) | 0.073 | 14 (8.2%) | 7 (8.3%) | 7 (8.3%) | 1.000 |
| Duration of ICU stay (day) | 3 (2,5) | 3 (2,4) | 3 (2,6) | ＜0.0001* | 3 (2,4) | 3 (2,5) | 3 (2,4) | 0.178 |
| In-hospital mortality (N,%) | 77 (11.2%) | 9 (5.3%) | 68 (13.2%) | 0.009* | 10 (4.8%) | 5 (4.8%) | 5 (4.8%) | 1.000 |

Non-normally distributed continuous variables are displayed as a median with interquartile range (IQR) and were compared using the Mann–Whitney U test. Categorical variables are expressed as counts with percentages and were compared using Pearson’s chi–square or Fisher’s exact test. Multiple samples are compared using the non-parametric Kruskal–Wallis test. Abbreviations: IBT: intraoperative blood transfusion; ALT: Alanine aminotransferase; AST: Aspartate aminotransferase; TB: Serum total bilirubin; EAD: early allograft dysfunction; AKI: acute kidney injury; RRT: renal replacement therapy; ICU: Intensive Care Unit.

*p-value <0.05.


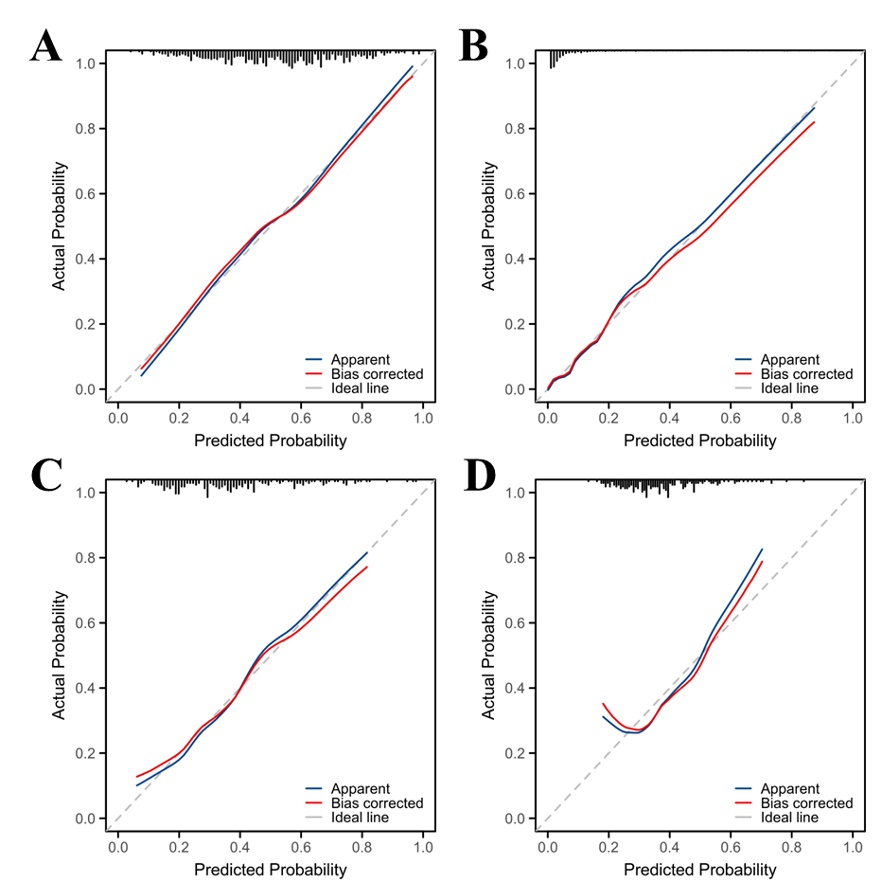


Figure S1. Calibration plots for multivariable logistic regression models in the unmatched and matched cohorts. Panels A–D show calibration curves comparing predicted and observed probabilities for the multivariable models. Panel A depicts the model for post‑LT AKI in the unmatched cohort (C‑index 0.729 [95% CI 0.688–0.770]; Hosmer–Lemeshow χ² = 3.8493, p = 0.8705), and panel B shows the model for in‑hospital mortality in the unmatched cohort (C‑index 0.877 [95% CI 0.819–0.934]; Hosmer–Lemeshow χ² = 7.739, p = 0.4594). Panel C presents the model for post‑LT AKI in the matched cohort (C‑index 0.748 [95% CI 0.677–0.818]; Hosmer–Lemeshow χ² = 6.1891, p = 0.6261), and panel D shows the model for in‑hospital mortality in the matched cohort (C‑index 0.644 [95% CI 0.562–0.727]; Hosmer–Lemeshow χ² = 8.3922, p = 0.3961). Across all four models, the C‑indices indicate acceptable to good discriminative ability, and non‑significant Hosmer–Lemeshow tests suggest adequate calibration.
